# Emphysematous cystitis with concomitant bilateral emphysematous ureteritis and pyelitis in a diabetic patient

**DOI:** 10.11604/pamj.2015.20.324.6657

**Published:** 2015-04-06

**Authors:** Fouad Hajji, Ahmed Ameur

**Affiliations:** 1 Department of Urology, Mohammed V Military University Hospital, Rabat, Morocco

**Keywords:** Emphysematous cystitis, emphysematous ureteritis, emphysematous pyelitis

## Image in medicine

A 70-year-old man presented to the Emergency Department with lower abdominal pain, abdominal distention and fever of 3 days duration associated with increased urinary frequency, urgency and pneumaturia. He was known case of type 2 diabetes mellitus on oral anti-diabetic agents for the past 15 years. On Physical examination, he had a temperature of 38, 8°C and a diffuse abdominal tenderness without any peritonism signs. Laboratory investigations showed leukocytosis (27500/µl), C-reactive protein 74 mg/l and serum creatinine 210 mmol /l. Urinalysis revealed pyuria and culture grew Escherichia Coli. Abdominal radiography (A, B, arrows) and computed tomography (C and D) revealed presence of gas within the wall and the lumen of the urinary bladder (UB), the ureters and the collecting systems. The patient was diagnosed as having emphysematous cystitis with concomitant bilateral emphysematous ureteritis and pyelitis. Given that there were no gas pockets or fluid collections seen within the renal parenchyma or in the perinephric tissues, emphysematous pyelonephritis was ruled out. Gas appearance inside the urinary system can be also caused by fistulas related to the gastrointestinal system, urinary system's interventional procedures, trauma or urethrally introduced objects (homosexuals). Our patient was treated with intravenous antibiotic, indwelling urinary catheter and insulin. Within 7 days, he had complete clinical and radiographical resolution of the gas in his urinary system, and his renal function returned to baseline. Emphysematous cystitis is a severe infection of the bladder due to gas-producing bacteria, often occurring in the patients with poorly controlled diabetes. Delayed diagnosis may lead to extension to the upper urinary tract, which seems to have happened in our patient.

**Figure 1:**
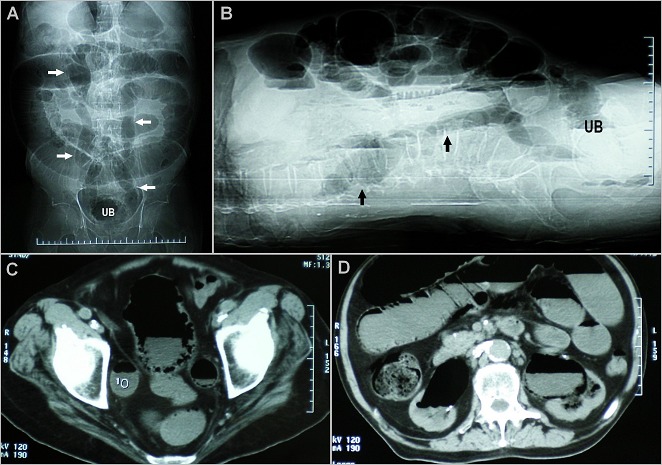
(A) supine frontal; (B) supine cross-table lateral abdominal radiographs showing linear air distribution along the collecting system of both kidneys, the ureters and around the urinary bladder (UB); (C) computerized tomography scan pelvis-sagittal section showing intramural air with intraluminal air-fluid level in the urinary bladder and the ureters; (D) computerized tomography scan abdomen-sagittal section showing intramural air with intraluminal air-fluid level in the pelvicalyceal systems. There were no gas pockets or fluid collections seen within the renal parenchyma or in the perinephric tissues

